# Longitudinal Changes in Peripheral Refraction, Lag and Axial Length in Myopic Children Treated with Orthokeratology: A 12-Month Prospective Study

**DOI:** 10.3390/children13081080

**Published:** 2026-08-14

**Authors:** Laura Batres, Julia Bodas-Romero, Cristina Arroyo-del Arroyo, María Serramito, Paloma Porras-Ángel, Patricia González-Diaz, Laura Valencia-Nieto, Gonzalo Carracedo

**Affiliations:** 1 Ocupharm Research Group, Department of Optometry and Vision, Faculty of Optics and Optometry, Complutense University of Madrid, 28037 Madrid, Spain; lbatres@ucm.es (L.B.); jbodas@ucm.es (J.B.-R.); mserrami@ucm.es (M.S.); palomapo@ucm.es (P.P.-Á.); patrgo16@ucm.es (P.G.-D.); lavalenc@ucm.es (L.V.-N.); 2 Instituto de Oftalmobiología Aplicada (IOBA), Department of Theoretical, Atomic and Optical Physics, Faculty of Science, University of Valladolid, 47011 Valladolid, Spain

**Keywords:** orthokeratology, peripheral refraction, accommodative lag, axial length, myopia control

## Abstract

**Highlights:**

**What are the main findings?**

- Orthokeratology treatment induces a stable and symmetrical pattern of relative peripheral myopic defocus along the horizontal meridian throughout a 12-month period.
- No statistically significant correlation was found between axial length and changes in peripheral refraction or accommodative lag in the overall sample.

**What are the implications of the main findings?**

- Monitoring peripheral refraction serves as a clinical validation of the lens-to-cornea relationship, confirming that the orthokeratology treatment is optically active.
- Children in the lag variation group during treatment demonstrated greater axial length increases than those in the no lag variation group. This exploratory finding requires confirmation in larger prospective studies.

**Abstract:**

**Background/Objectives**: High accommodative lag and relative peripheral hyperopic defocus during near work have been proposed as potential contributors to myopia progression. Orthokeratology (OK) reshapes the cornea to provide central correction while inducing peripheral myopic defocus. This prospective 12-month study aimed to evaluate longitudinal changes in peripheral refraction, accommodative lag, and axial length (AL) in myopic children treated with OK. **Methods**: Children aged 7 to 17 years with low-to-moderate myopia were fitted with spherical (CRT™ STD) or toric (CRT™ DA) OK lenses and followed for 12 months. Horizontal peripheral refraction (vector M, J0, J45) up to ±30°, accommodative lag and non-cycloplegic AL were measured. **Results**: Forty-seven participants completed the study. Mean AL increased from 24.76 ± 0.87 mm at baseline to 24.86 ±0.85 mm at 12 months (*p* < 0.001). OK induced a stable, symmetrical redistribution of relative peripheral myopic defocus across visits. The toric design showed more consistent significant variations in the J0 astigmatic component across eccentricities. No significant correlations were found between AL elongation and changes in peripheral refraction (*p* = 0.194) or accommodative lag (*p* > 0.05) in the overall sample. However, subjects in the lag variation group showed significant AL increases (*p* < 0.05), while those in the no lag variation group did not (*p* > 0.05). **Conclusions**: OK consistently induces a stable pattern of peripheral myopic defocus. While peripheral defocus and accommodative lag do not linearly correlate with global axial growth, accommodative behavior may be associated with individual differences in axial growth. Biometry remains important for monitoring treatment outcomes.

## 1. Introduction

Orthokeratology (OK) lenses cause a flattening of the central area and a curving of the mid-periphery on the anterior surface, increasing the refractive power of the cornea. This central shape corrects the refractive error providing good visual acuity, while the periphery appears to be responsible for inducing relative myopic blur on the peripheral retina, which is considered one of the mechanisms by which OK helps control myopia. Compared to single-vision spectacles and contact lenses, OK is estimated to slow axial eye growth by approximately 32–63% per year, depending on ethnicity and age group [1].

Accommodation during near work is considered a major factor in myopia development. It may induce accommodative lag, defined as under-accommodation that results in hyperopic retinal defocus in the central visual field [2].

To evaluate treatment efficacy, most studies assess both changes in axial length (AL)—or alternatively vitreous chamber depth—using optical or ultrasound biometry, and changes in peripheral refraction, typically expressed as the power vectors M, J0, and J45 measured along the horizontal visual field under open-field conditions [3,4,5]. The peripheral spherical equivalent (M) varies across the horizontal meridian after OK treatment, with the greatest myopic defocus observed in the nasal retina. This asymmetric profile has been attributed to the well-described nasal-temporal asymmetry of the eye, the temporal decentration of OK lenses, and differences in corneal curvature and elevation between the nasal and temporal cornea. Peripheral astigmatism is also modified following OK. Specifically, the J0 component becomes more negative in both the nasal and temporal retina, although the nasal retina continues to exhibit the most negative values, thereby maintaining the previously described asymmetry. In contrast, changes in the J45 component are less consistent and generally have not been found to be statistically significant [3,6,7,8].

Recent evidence suggests that the effect of OK on ocular growth and refractive changes may depend on baseline refractive status, although its relationship with myopia progression remains unclear. Gifford et al. [8] reported that peripheral refractive changes induced by OK vary according to the degree of myopia, but these changes were not associated with myopia progression. Similarly, the LORIC study by Cho et al. [9] found a significant positive correlation between baseline spherical equivalent (SE) and changes in vitreous chamber depth, indicating a smaller increase—and therefore slower myopia progression—in subjects with higher initial myopia when compared with spectacle wearers. Kakita et al. [10] also observed a significant association between baseline SE and axial elongation, showing that highly myopic subjects exhibited less axial growth over a 2-year period in the OK group, a relationship that was not observed in the spectacle-wearing control group. In contrast, Zhu et al. [11] reported that axial elongation was related to age at myopia control intervention and spectacle wear, but not to sex, baseline refractive error, initial AL, or baseline corneal curvature.

The clinical role of peripheral refraction in myopic progression remains unclear and controversial. In OK, changes in the anterior corneal curvature are closely related to the amount of myopia to be corrected; these changes in anterior corneal power are thought to influence the retinal defocus profile and AL [12]. Therefore, as Zhong et al. [13] suggested, it is to be expected that the greater the induced paracentral corneal power, the greater the amount of myopia induced in retina periphery, the greater control over AL growth. However, one study warned that OK treatment does not guarantee peripheral myopization in all degrees of myopia because corneal changes in low myopia are insufficient [5]. Moreover, Santodomingo-Rubido et al. [14] reported in 2018 that the reduction in central corneal power and the relative increase in the paracentral and pericentral zones induced by OK after 2 years of treatment are not significantly correlated with changes in AL in White European children.

In summary, although numerous studies have demonstrated changes in peripheral refraction following OK, few have investigated whether these changes may be influenced by alterations in accommodative status in children wearing OK lenses. Therefore, the aim of this study was to assess peripheral refraction along the horizontal meridian and to analyze its relationship with AL changes and accommodative lag, in order to better understand the potential role of peripheral defocus changes in myopia control.

## 2. Materials and Methods

### 2.1. Study Design and Sample

This prospective, randomized and longitudinal study was conducted in accordance with the principles of the Declaration of Helsinki and Good Clinical Practice guidelines and was approved by the Ethics Committee of Hospital Clínico San Carlos (Madrid, Spain). Written informed consent was obtained from all participants and from their parents or legal guardians prior to enrolment.

Participants were followed for 12 months and attended eight scheduled visits: baseline (before orthokeratology fitting) (PRE-OK), after the first night of lens wear (1N), and at 1 week (1W), 1 month (1M), 3 months (3M), 6 months (6M), and 12 months (12M) of treatment, followed by a final assessment one month after treatment discontinuation (POST-OK). Eligible participants were children aged 7–17 years with non-cycloplegic myopia between −0.50 and −6.00 diopters (D) and astigmatism below 2.50 D at baseline. Individuals with a history of ocular inflammation or ocular disease were excluded from the study.

One participant had one eye that slightly exceeded the upper myopia inclusion limit (−7.75 D), whereas the fellow eye fulfilled all eligibility criteria. Both eyes were retained in the analysis to preserve the paired-eye design of the study. Following the one-month post-treatment discontinuation period, this eye reached −8.25 D, consistent with approximately −0.50 D of natural myopic progression over the 12-month follow-up.

All measurements were taken under normal conditions, without the use of cycloplegic.

### 2.2. Lens Fitting

All participants were fitted with Corneal Refractive Therapy (CRT™) orthokeratology lenses (Paragon Vision Sciences, Gilbert, AZ, USA) manufactured in HDS 100 material (Paflufocon D; Dk = 100 barrer), following the manufacturer’s fitting recommendations. The design for the initial lens for the right and left eyes of the patient was selected using a random calculation system with Microsoft Excel 2000 (Microsoft Corporation, Redmond, WA, USA). Variable 1 was assigned to the CRT™ standard design (STD), and variable 2 was assigned to the CRT™ Dual Axis design (DA). Changes in lens parameters and even in lens design, from STD to DA, were made based on fluorogram pattern and visual acuity, especially if there was incomplete lens landing across the cornea (360°).

The CRT™ lens incorporates a reverse-geometry design composed of three functional zones: the base curve (optical zone, OZ), the Return Zone Depth (RZD), and the Landing Zone Angle (LZA). Depending on the corneal characteristics, either the STD or DA design was prescribed. The DA design incorporates two meridians with different sagittal heights to improve lens centration on toric corneas, whereas the STD design is intended for more regular corneal geometries.

Instructions on wearing, insertion, removal and care system (Menicare Pure (Menicon, Nagoya, Japan), Progent (Menicon, Nagoya, Japan) and Lacrifresh ocu-dry artificial tears (AVIZOR, Madrid, Spain)) were given to the subjects.

### 2.3. Peripheral Refraction

Peripheral refraction was measured following the procedure described by Queirós et al. [3]. Measurements were obtained monocularly using an open-field autorefractometer (Shin-Nippon SRW-5000, Grand Seiko Co., Ltd., Hiroshima, Japan). Subjects were positioned 2.5 m from the fixation target. Room illumination was adjusted to maintain a pupil diameter of at least 4 mm, ensuring reliable peripheral refraction measurements.

A curved platform was mounted in front of the autorefractometer and equipped with illuminated fixation targets positioned at eccentricities of 5°, 10°, 15°, 20°, 25° and 30° in the nasal and temporal direction. The assembly instructions are detailed below: The chord of the arc between the two outer LEDs (at 35° nasal and 35° temporal) is 186 cm. The LEDs emit white light and were 5 mm of diameter. They were point light sources whose brightness can be adjusted using a dimmer. The height of the letter E was 28 mm, and the minimum detail of the E was 3 mm. For a distance of 2.5 m, the corresponding visual acuity was 0.2 (decimal) or 0.7 (LogMAR). It was made using 15 LEDs and 15 letters E of the same size. These letters and lights were placed at the following angles: 0°, 5°, 10°, 15°, 20°, 25°, 30°, 35° to the right and the same angles to the left. Measurements were taken to 30 degrees due to the size of the outer frame of the open-field autorefractometer’s window.

For the test, the subject was required to keep their head still and look straight ahead. They had to follow the stimulus placed on the platform at 2.5 m away while the examiner determined the refraction by analyzing the image of an infrared light ring reflected from the retina to the cornea. Cycloplegia was not used for the measurements along horizontal meridian at 0°, 5°, 10°, 15°, 20°, 25°, 30° in the nasal and temporal directions. Three measurements were taken in each eye with the reticle centered and focused on subject’s pupil.

Spherical, cylinder and axis values were recorded by the autorefractometer’s software. These data were transcribed into an Excel spreadsheet, where it was turned into different vectors M, J0 and J45. M = sphere + cylinder/2, J0 = −(cylinder/2) × cos (2 × axis), J45 = −(cylinder/2) × sin (2 × axis).

### 2.4. Biometry

AL measurements were taken using the Aladdin Optical Biometer (Topcon, Tokyo, Japan) under normal conditions, without the use of cycloplegic. For statistical analysis, the mean value indicated by the device for each measurement was used. It was considered that if, after a year, axial growth was less than 0.30 mm, control of the subject’s myopic progression had been achieved [15].

### 2.5. Accommodative Lag Assessment

Accommodative response was assessed using Nott retinoscopy. Measurements were performed binocularly at a viewing distance of 40 cm while participants wore their full distance refractive correction, if required. An isolated ETDRS near visual acuity target (0.8 decimal visual acuity) was used as the accommodative stimulus. Following the procedure described by Stark and Atchison [16] the examiner determined the neutralization point of the retinoscopic reflex using a Heine Beta 200 retinoscope (Heine, Herrsching, Germany). Neutralization occurring behind the stimulus plane was recorded as accommodative lag, whereas neutralization occurring in front of the stimulus plane was considered accommodative lead. Three measurements were obtained for each eye, and the mean value was used for statistical analysis.

### 2.6. Statistical Analysis

The data were analyzed using the SPSS statistical package version 22.0 for Windows (SPSS, Inc., Chicago, IL, USA) and Microsoft Office Excel (Microsoft Corporation, Redmond, WA, USA). All measurements were averaged across both eyes except for peripheral refraction when OK designs were compared (contralateral paired-eye) to perform the statistical analysis.

Student’s *t*-test for related samples using the Benjamini–Hochberg False Discovery Rate (FDR) adjustment was used to compare peripheral refraction in the different eccentricities among visits. Subject-level covariates (age, sex, baseline spherical equivalent, and parental myopia) were inherently controlled by the within-subject variance of the contralateral study design.

Repeated measures ANOVA was used to assess the AL differences over the course of the treatment period and Student’s *t*-test for related samples using the Bonferroni correction was used to compare the baseline visit with the follow-up OK visits.

Changes in AL were also assessed according to the variation in accommodative lag during treatment. Subjects were classified into two groups: the lag variation group (subjects who showed a decrease in accommodative lag between baseline and the end of the study period) and the no lag variation group (subjects who did not show a decrease in accommodative lag). To analyze the correlation between AL, accommodative lag and peripheral refraction, Pearson’s’s correlation coefficients were used, and the linear trend equation was calculated using linear regression analysis. For the correlation analysis, three variables were defined for the 12-month analysis: (A) AL change, calculated as the difference between AL at each visit and baseline (A = AL_visit − AL_pre); (B) peripheral refraction change, derived from the RPRE at 30° eccentricity (mean of nasal and temporal retina) with respect to the central value at each visit, and subsequently expressed as the difference from baseline (B = B_visit − B_PRE-OK); and (C) accommodative lag change, defined as the difference between accommodative lag at each visit and its baseline value (C = lag_visit − lag_pre).

Values are expressed as Mean ± SD (standard deviation). Results were considered statistically significant when *p* < 0.05.

## 3. Results

### 3.1. Baseline Demographics

A total of 55 participants were recruited, and 47 participants completed all scheduled follow-up visits over the one-year study period. Of the 8 participants who dropped out of the study, 5 were for bad vision or/and handling and 3 due to lens design changes from DA to STD. At baseline, the mean SE refractive error was −3.23 ± 1.57 D, ranging from −7.75 to −1.00 D. The mean age of the cohort was 12.43 ± 2.46 years, with participants aged between 8 and 17 years. Forty-two subjects also attended the visit conducted one month after discontinuation of lens wear. At this point, the mean SE refractive error was −3.23 ± 1.48 D, with values ranging from −8.25 to −1.00 D.

Regarding optometric history, 43 participants reported a progression of myopia within the two years preceding study enrollment. Among them, 12 had a family history of myopia affecting both parents. In terms of visual correction prior to the study, seven participants were habitual wearers of soft contact lenses, whereas the remaining 40 subjects relied exclusively on spectacle correction.

### 3.2. Longitudinal Changes in AL

AL measurements were obtained at baseline (PRE-OK) and during follow-up visits throughout the 12-month treatment period.

Mean AL showed a gradual increase over the study period. Baseline values were 24.76 ± 0.87 mm, followed by 24.66 ± 0.90 mm at 1 month, 24.72 ± 0.85 mm at 3 months, 24.78 ± 0.86 mm at 6 months, and 24.86 ± 0.85 mm at 12 months. The overall change across time points was statistically significant (*p* = 0.032; ANOVA for related samples).

Comparisons indicated that AL at 12 months was significantly greater than at baseline (*p* < 0.001; *t*-test for related samples). However, no significant differences were observed between baseline and the intermediate follow-up visits at 1 month (*p* = 0.072; *t*-test for related samples), 3 months (*p* = 0.089; *t*-test for related samples), or 6 months (*p* = 0.211; *t*-test for related samples).

### 3.3. Peripheral Refraction Profile

The changes in relative peripheral refractive error (RPRE) were assessed across the ±30° visual field (total 60°), and expressed using vector components M, J0 and J45. RPRE was calculated as the difference between the refractive error measured at each eccentric location and the central refractive value (0°).

**Figure 1:**
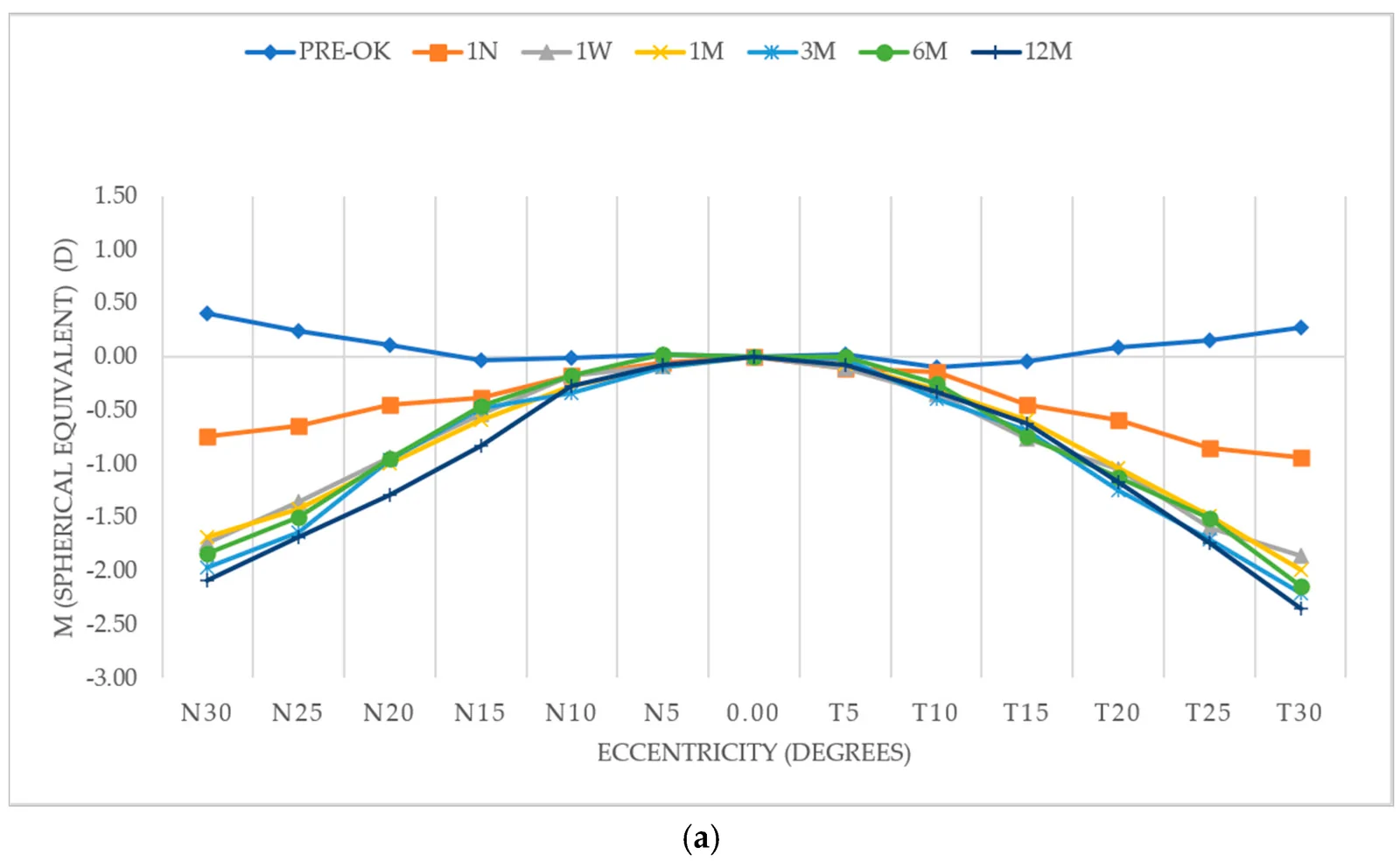

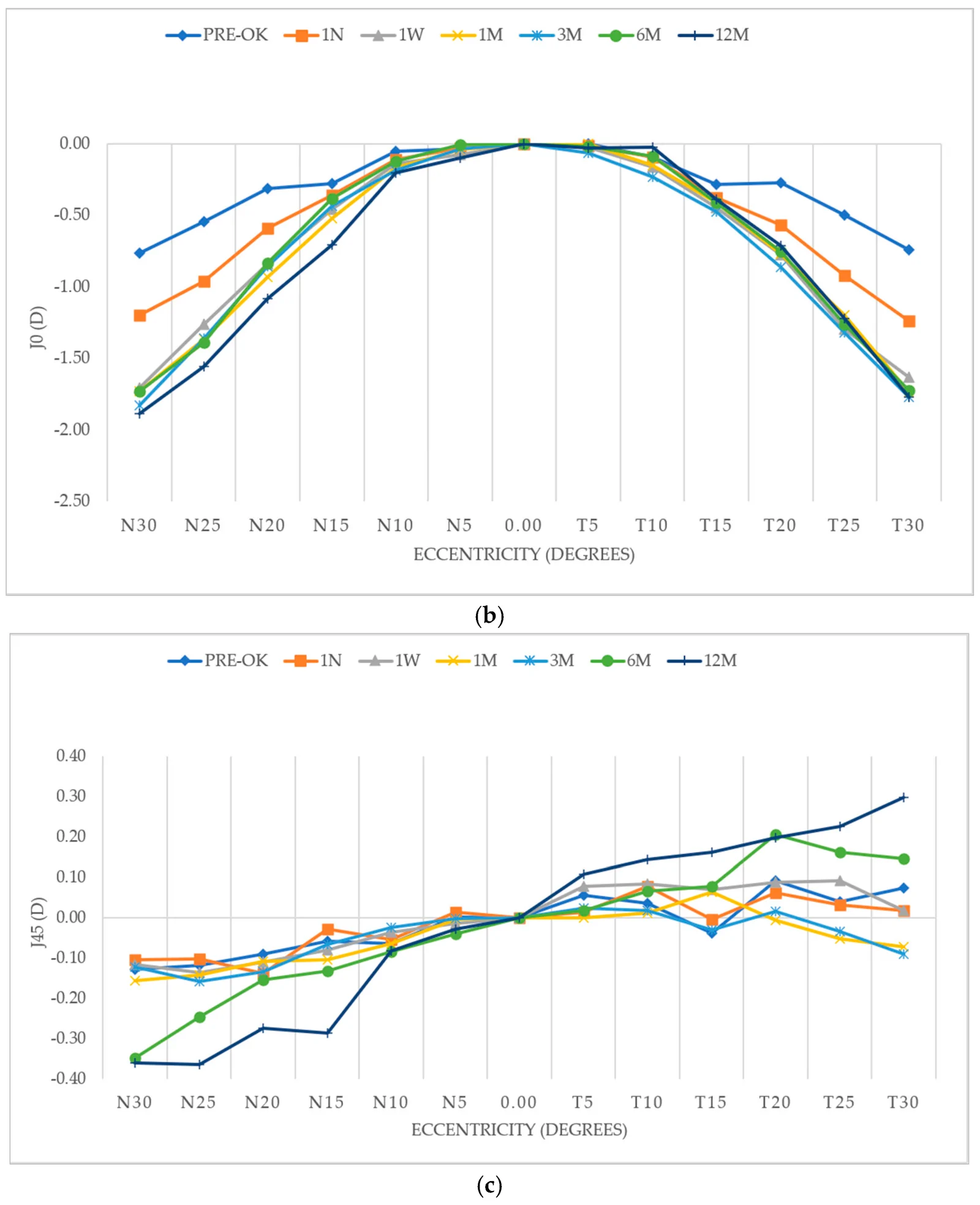
Relative peripheral refractive error (RPRE) plots of the vector components: M (**a**), J_0_ (**b**), and J_45_ (**c**) for the entire sample. Mean RPRE at baseline (PRE-OK) and at each visit: 1N (first night), 1W (first week), 1M (first month), 3M (three months), 6M (six months), and 12M (twelve months) for the total sample of subjects fitted with orthokeratology lenses. The plots represent peripheral defocus normalized to the central value (0°) to allow comparison across all graphs. N: nasal; T: temporal; D: diopters.

Figure 1 illustrates the distribution of M, J0 and J45 as a function of visual field angle in both temporal and nasal retinal regions. Measurements are presented for all time points (PRE-OK, 1N, 1S, 1M, 3M, 6M, and 12M) considering the total sample. As shown in the figure, OK induced a symmetrical pattern of relative peripheral defocus between the temporal and nasal retinal areas. This pattern was consistently observed across visits, indicating a stable bilateral and symmetrical redistribution of peripheral refractive error characterized by changes in peripheral optics while maintaining central refractive correction throughout the follow-up period.

Further analysis was conducted by stratifying the data according to the OK lens design (CRT™). The RPRE profiles were evaluated separately for eyes fitted with CRT™ STD and CRT™ DA lenses.

For the spherical CRT™ STD design, Table 1 summarizes the visual field locations where statistically significant changes were observed (*p* < 0.05), as determined by post-hoc paired Student’s *t*-tests adjusted using the Benjamini–Hochberg False Discovery Rate (FDR) procedure. The corresponding mean values and standard deviations for peripheral refraction (M, J0 and J45) at each eccentricity and follow-up visit for both lens designs are provided in Appendix A.

For the toric CRT™ DA design, Table 2 presents the *p*-values corresponding to the statistical comparisons of the peripheral refractive components at each visual field angle. Overall, statistically significant differences—after controlling for multiplicity via FDR adjustment—were observed across a wider range of angles and time points compared to the spherical design. The SE component (M) showed consistent significant changes across most peripheral locations from early visits, indicating a pronounced and stable induction of peripheral myopic defocus. Similarly, the astigmatic component J0 exhibited significant variations across multiple angles, particularly in both nasal and temporal retina, while J45 showed fewer significant changes at specific locations.

Reversibility of the induced peripheral refractive changes was evaluated by analyzing measurements obtained one month after discontinuation of OK lens wear. For the total sample, no statistically significant differences were observed in the RPRE for the vector components M, J0, and J45 at any of the evaluated angles when compared POST-OK to PRE-OK. The only exception was found at 10° in the temporal retina, where the J0 component showed a small but statistically significant increase compared with PRE-OK values (*p* = 0.039; paired Student’s *t*-test). When the analysis was performed according to lens design, the CRT™ DA subgroup showed statistically significant increases in the J0 component at 5° nasal, 5° temporal, 10° temporal, and 15° temporal (*p* < 0.05; paired Student’s *t*-test).

### 3.4. Correlation Analysis

#### 3.4.1. Correlation AL Change and Peripheral Refraction

The relationship between axial elongation and changes in RPRE was analyzed to investigate their potential association during OK treatment. AL variation was defined as the difference between the 12-month and baseline measurements, while peripheral refraction change was calculated from the variation in RPRE between the same time points.

Correlation analysis was performed using Pearson’s’s correlation coefficient (r). At 12 months, a weak positive correlation was observed between AL change and peripheral refractive error variation (r = 0.193). However, this association did not reach statistical significance (*p* = 0.194; Pearson’s correlation). Despite the progressive axial elongation and the consistent modification of the peripheral refractive profile, the relationship between both variables was limited.

#### 3.4.2. Correlation AL Change and Accommodative Lag

**Figure 2:**
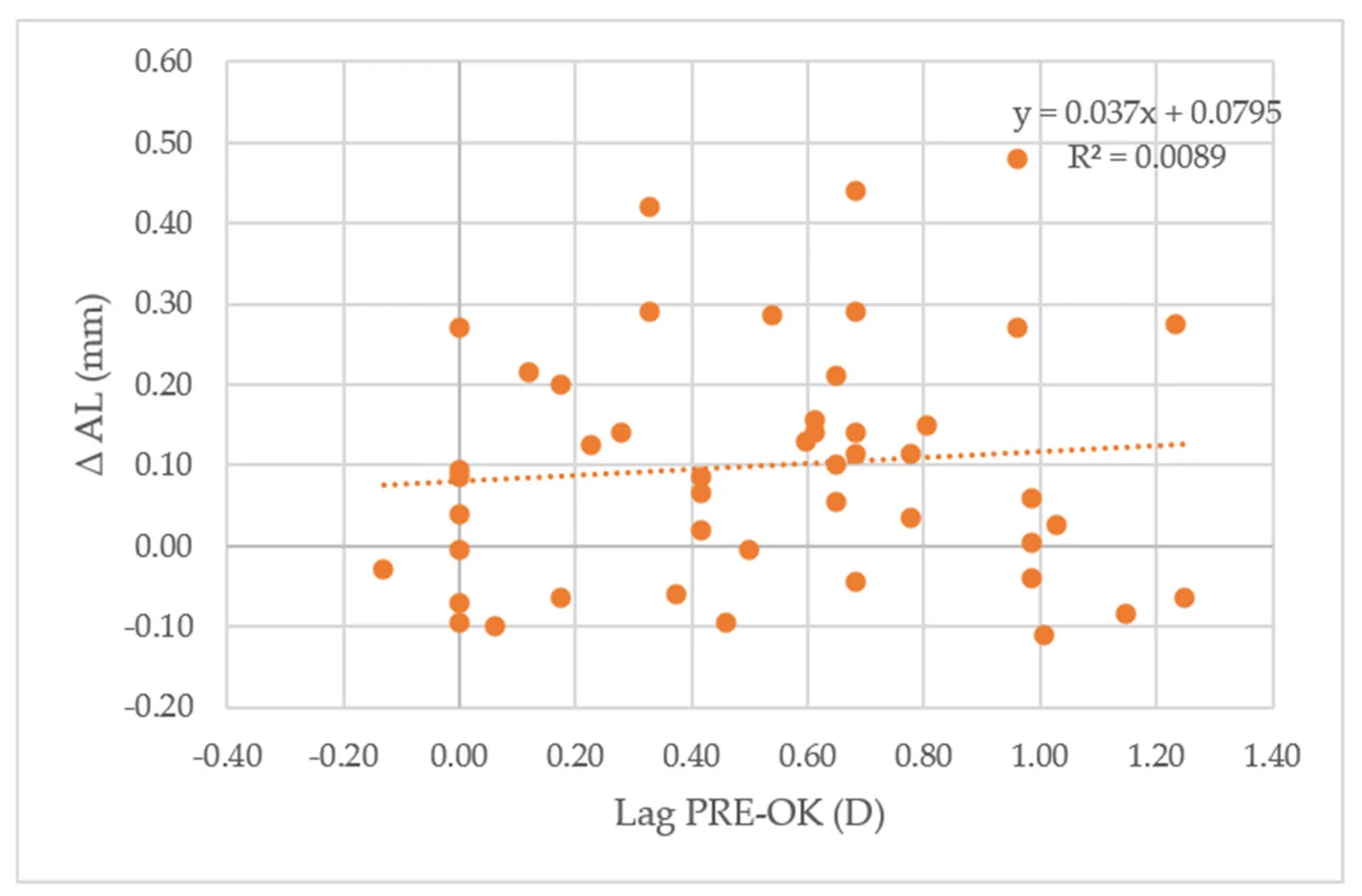
Scatter plot illustrating the relationship between baseline accommodative lag (Lag PRE-OK, D) and axial length (AL) change (ΔAL, mm) during orthokeratology (OK) treatment. Each point represents an individual subject. The dashed line corresponds to the linear regression fit (y = 0.037x + 0.0795), showing a very weak association between variables (R^2^ = 0.0089). D: diopters.

Mean values and standard deviations for the accommodative lag during OK are provided in Appendix A. The relationship between axial elongation and accommodative lag was analyzed using Pearson’s correlation. As illustrated in Figure 2, the relationship between baseline accommodative lag and subsequent AL change was negligible, with a very weak correlation (R = 0.094).

When considering changes over time, no meaningful correlation was observed between AL variation and accommodative lag change in the overall sample. However, in a subgroup of participants exhibiting greater axial elongation (>0.30 mm), a moderate positive correlation (r = 0.633) was identified between AL and accommodative lag after treatment cessation. During OK wear, these subjects showed a reduction in accommodative lag, which partially reversed following lens discontinuation.

Further subgroup analysis indicated that participants included in the lag variation group showed statistically significant increases in AL from the 3-month visit onward (*p* < 0.05; paired Student’s *t*-test), whereas no significant AL changes were detected in the no lag variation group (*p* > 0.05) (Table 3).

A subset of the study cohort (*n* = 5) exhibited an AL increase greater than 0.30 mm, with a mean increase of 0.43 ± 0.13 mm. In these specific subjects, mean changes in accommodative lag were observed, corresponding to an accommodative lead of −0.78 ± 0.52 D.

#### 3.4.3. Correlation AL Change, Peripheral Refraction, and Accommodative Lag

The relationship between axial elongation, relative peripheral refractive changes, and accommodative lag was evaluated to explore their combined role in myopia progression. Baseline values and methodological details regarding accommodative lag assessment have been previously described in a companion study by Batres et al. [17].

As shown in Table 4, after 12 months of OK treatment, a weak positive correlation was found between AL change and peripheral refraction change. However, none of these associations reached statistical significance (*p* > 0.05; Pearson’s correlation analysis).

Following one month of OK lens discontinuation, AL exhibited a weak negative correlation with accommodative lag (Table 5). Similarly, peripheral refraction change was negatively correlated with accommodative lag. In contrast, the association between AL and peripheral refraction was positive, although very weak in magnitude. Overall, the correlations observed after treatment cessation were low, indicating limited interaction among axial elongation, peripheral refractive changes, and accommodative response once lens wear had been discontinued.

## 4. Discussion

One of the proposed mechanisms underlying myopia progression suggests that relative hyperopic defocus in the peripheral retina produced by a high accommodative lag during near work may stimulate axial elongation of the eye [18,19]. Nevertheless, the evidence remains inconclusive, as studies have reported conflicting findings regarding whether accommodative lag acts as an independent predictor of myopia progression or simply accompanies, rather than precedes, the onset of the condition [20,21,22,23]. The present study investigated the relationship between AL, RPRE, and accommodative lag in a pediatric population undergoing OK contact lens wear for myopia control after a one-year follow-up treatment. Accommodative function does not appear to contribute to the peripheral optical profile induced by treatment. Although it may be weakly associated with axial elongation, its role in the mechanisms underlying myopia control appears to be limited.

The results found in this study revealed a significant increase in AL of 0.10 ± 0.15 mm at the end of the follow-up period. This value was lower than those reported in other studies using the same contact lens designs, in which AL increases ranging from 0.18 ± 0.24 mm to 0.27 ± 0.15 mm were observed, maybe due to the characteristics of the study sample [24,25]. For this reason, the subjects were further subdivided according to whether a change in accommodative lag was observed. This division revealed a significant increase in AL among subjects in the lag variation group (AL increased from 24.76 ± 0.80 mm PRE-OK to 24.90 ± 0.77 mm at month 12; *p* < 0.001; *n* = 36), whereas no significant increase in AL was observed in the no lag variation group (AL PRE-OK: 24.69 ± 1.13 mm; AL at month 12: 24.73 ± 1.13 mm; *p* = 0.385; *n* = 11). This finding partially aligns with the results found in a previous study [26], where, during the first 6 months of treatment, a better accommodative response was associated with less axial elongation. But after 12 months of treatment, this association was no longer statistically significant. These findings could suggest that accommodative function may be associated with axial elongation during OK treatment, although this relationship remains uncertain.

As expected, in this study, OK induced a stable pattern of peripheral myopic defocus (primarily reflected in M), which was maintained over the long term. The whole sample exhibited a relatively symmetric pattern in the relative defocus generated between the nasal-temporal quadrants. When comparing peripheral refraction patterns between subjects fitted with the spherical and toric contact lens designs, no differences were found in terms of the M component. However, differences were observed in the J0 component, with significant *p* values being more present at each eccentricity and at each visit in subjects fitted with the toric design. This may be explained by the better lens centration observed in subjects fitted with toric contact lens designs throughout the treatment period (Clinical evaluation; data not provided). Lens decentration has already been associated with asymmetric relative peripheral refraction changes in the horizontal quadrants. It has been observed that the majority of decentrations occur in the inferotemporal quadrant, leading to a greater myopic shift in the temporal retina than in the nasal side [27,28]. To the best of the authors’ knowledge, no published studies have directly compared peripheral refraction between spherical and toric OK designs. While some authors [29] have reported improved lens centration with toric peripheral designs, peripheral refraction was not evaluated. Therefore, the present findings provide novel evidence regarding the influence of lens design on peripheral refractive changes.

No significant correlation was found between AL and peripheral refraction after one year of OK treatment. One possible explanation is that peripheral refraction measurements may have been influenced by the testing distance, accommodative responses, and the participant’s ability to maintain fixation on the designated target. Additionally, AL measurements were obtained without cycloplegia, which may have contributed to measurement variability. Although recent studies have reported associations between regional peripheral myopic defocus and axial elongation during OK treatment, these relationships appear to be location-dependent and not consistently observed across all retinal areas. One study found no significant associations between axial elongation and changes in the relative peripheral refraction across the overall 53° retinal region analyzed. However, significant associations were found within the 30–40° region and in the inferior retina [30]. Similarly, another study reported significant associations between axial elongation and several regional retinal defocus values, including measurements at 53° and 45°, as well as across the 30–45° and 45–53° regions, whereas no significant associations were observed at 15° and 30°, or across the 15–30° region [31]. Nevertheless, the low magnitude of the correlation found was only weak to moderate (r = 0.30–0.45), suggesting that changes in RPRE are region-dependent rather than global and may not be directly associated with axial growth within the sample studied over the 12-month period.

Although differences in AL growth were observed between the lag variation and no lag variation groups, the association between AL and accommodative lag was weak and not statistically significant in the overall sample and in both subgroups. These results are consistent with those found in a previous study evaluating OK wear over 9 months, which reported no significant association between AL and accommodative function [32], further supporting the lack of a consistent relationship between these variables. The role of accommodation in myopia control remains relatively underexplored and not yet fully understood, but it seems that accommodative changes could be unlikely to play a major role in myopia control mechanisms. Further investigation to clarify its potential contribution and interaction with other factors involved in axial elongation is warranted.

One month after discontinuing OK, myopic defocus returned to near baseline conditions and no significant correlations between AL, relative peripheral refraction, and accommodative lag were observed. In those subjects who exhibited an AL increase greater than 0.30 mm, changes in accommodative lead observed after 12 months of treatment of −0.78 ± 0.52 D reverted to −0.21 ± 0.26 D after one month without lens use. In addition, a moderate correlation between lag variation POST-OK and AL POST-OK was observed (r = 0.633). There is limited evidence specifically addressing the reversibility of peripheral refractive profiles or accommodative change after OK cessation. But in the present study, the reversibility of accommodative lag changes after discontinuation of OK may suggest that these alterations are primarily related to the optical and functional effects induced by the treatment. In this context, patients in the lag variation group during OK for myopia control also showed greater axial elongation and may therefore require closer monitoring or additional management strategies aimed at improving accommodative function. Although this finding may suggest a potential association, it should be interpreted with caution.

Clinical Implications for Pediatric Practice:

Our results confirm that both Paragon CRT™ STD and DA designs consistently induce a relative peripheral myopic defocus along the horizontal meridian. The statistical significance observed aligns with the optical theory of OK, where the redistribution of the corneal epithelium creates a mid-peripheral plus power ring. From a clinical perspective, this confirms that the treatment is optically active. No significant correlation was found between the magnitude of peripheral M-vector change and axial elongation (r = 0.193), suggesting that peripheral defocus alone does not determine the myopia control response. These findings support the hypothesis of a threshold effect, whereby additional peripheral myopic defocus may not provide proportional benefits once axial growth is adequately controlled.

The observation that peripheral refraction returns to baseline levels upon treatment cessation is of critical clinical importance. It suggests that the peripheral myopic defocus induced by orthokeratology—and its proposed myopia-control effect according to the peripheral defocus theory—is transient and purely dependent on active lens wear.

Although the findings of this study provide valuable insights into the relationship between peripheral refraction, lag and AL in myopia control, several limitations should be acknowledged when interpreting the results. First, no control group was included, as myopia control was not the primary objective of this study. Second, only participants with low to moderate myopia were enrolled; therefore, the effects of accommodation on AL and peripheral defocus in highly myopic individuals remain unclear. Third, because accommodation is a binocular process, it was not possible to determine whether differences in peripheral lens sagittal depth influenced the myopia control response. A further limitation of this study is that pupil diameter was not measured during peripheral refraction assessments. Although all examinations were performed under standardized testing conditions, including consistent illumination and fixation procedures to minimize variability, the potential influence of physiological changes in pupil size on peripheral refraction measurements cannot be completely excluded.

## 5. Conclusions

This 12-month prospective study showed that OK induces a stable peripheral myopic defocus pattern that was maintained throughout treatment, while AL exhibited a modest but significant increase over the follow-up period. Although greater axial elongation was observed in subjects in the lag variation group compared with those in the no lag variation group neither accommodative lag nor peripheral refraction was significantly correlated with AL change, suggesting that these factors were not directly related to axial growth during OK treatment. The reversibility of these factors after treatment cessation may support their functional and treatment-dependent nature. These findings highlight a potential interaction between accommodative behavior and axial elongation during OK wear, although the underlying relationship appears to be complex and not directly mediated by peripheral refraction alone. However, this observation should be interpreted with caution given the limited sample size and the absence of a control group. Further longitudinal studies are needed to clarify the relationship between accommodative function and axial growth during orthokeratology treatment.

## Figures and Tables

**Table 1:** Statistical significance values (*p*-values) for the vector components of peripheral refraction (M, J0, and J45) for the CRT™ STD design.

	30N	25N	20N	15N	10N	5N	0	5T	10T	15T	20T	25T	30T
1N	J_0_ *p* < 0.001	J_0_ *p* = 0.004	J_0_ *p* = 0.021		M *p* = 0.017	M *p* = 0.004	M *p* = 0.03	M *p* = 0.017	M *p* = 0.034		J_0_ *p* = 0.002	J_0_ *p* < 0.001	J_0_ *p* = 0.001
1W	J_0_ *p* < 0.001	J_0_ *p* < 0.001	J_0_ *p* < 0.001	M *p* = 0.001	M *p* < 0.001	M *p* < 0.001	M *p* < 0.001	M *p* < 0.001	M *p* < 0.001	M *p* = 0.003	J_0_ *p* < 0.001	J_0_ *p* < 0.001	J_0_ *p* < 0.001
					J_0_ *p* = 0.030				J_0_ *p* = 0.032	J_0_ *p* = 0.001			
1M	J_0_ *p* < 0.001	M *p* = 0.037	M *p* = 0.001	M *p* < 0.001	M *p* < 0.001	M *p* < 0.001	M *p* < 0.001	M *p* < 0.001	M *p* < 0.001	M *p* < 0.001	M *p* < 0.001	M *p* = 0.003	J_0_ *p* < 0.001
		J_0_ *p* < 0.001	J_0_ *p* < 0.001	J_0_ *p* = 0.005	J_0_ *p* = 0.047						J_0_ *p* = 0.001	J_0_ *p* < 0.001	
3M	J_0_ *p* < 0.001	J_0_ *p* < 0.001	M *p* = 0.002	M *p* < 0.001	M *p* < 0.001	M *p* < 0.001	M *p* < 0.001	M *p* < 0.001	M *p* < 0.001	M *p* < 0.001	J_0_ *p* < 0.001	J_0_ *p* < 0.001	J_0_ *p* < 0.001
			J_0_ *p* < 0.001						J_0_ *p* = 0.052	J_0_ *p* = 0.032			
6M	J_0_ *p* < 0.001	J_0_ *p* < 0.001	M *p* = 0.009	M *p* < 0.001	M *p* < 0.001	M *p* < 0.001	M *p* < 0.001	M *p* < 0.001	M *p* < 0.001	M *p* < 0.001	J_0_ *p* = 0.005	J_0_ *p* < 0.001	J_0_ *p* < 0.001
	J_45_ *p* = 0.017		J_0_ *p* = 0.003										
12M	J_0_ *p* < 0.001	J_0_ *p* < 0.001	J_0_ *p* < 0.001	J_0_ *p* = 0.004	M *p* < 0.001	M *p* < 0.001	M *p* < 0.001	M *p* < 0.001	M *p* < 0.001	M *p* < 0.001	M *p* = 0.024	J_0_ *p* < 0.001	J_0_ *p* < 0.001
	J_45_ *p* = 0.019	J_45_ *p* = 0.005	J_45_ *p* = 0.015	J_45_ *p* = 0.009							J_0_ *p* = 0.001		J_45_ *p* = 0.050

Only statistically significant *p*-values are displayed in the matrix. For each visual field angle, *p*-values correspond to paired comparisons between baseline and each follow-up visit using Student’s *t*-test for related samples (*p* < 0.05). All reported *p*-values have been adjusted for multiple comparisons across eccentricities and visits using the Benjamini–Hochberg False Discovery Rate (FDR) procedure to control for Type I errors.

**Table 2:** Statistical significance values (*p*-values) for the vector components of peripheral refraction (M, J0, and J45) for the CRT™ DA design.

	30N	25N	20N	15N	10N	5N	0	5T	10T	15T	20T	25T	30T
1N	J_0_ *p* = 0.002	J_0_ *p* < 0.001	J_0_ *p* = 0.001	M *p* = 0.042	M *p* = 0.008	M *p* = 0.004	M *p* < 0.001	M *p* = 0.003	M *p* = 0.001		J_0_ *p* = 0.002	J_0_ *p* < 0.001	J_0_ *p* < 0.001
1W	J_0_ *p* < 0.001	J_0_ *p* < 0.001	M *p* < 0.001	M *p* < 0.001	M *p* < 0.001	M *p* < 0.001	M *p* < 0.001	M *p* < 0.001	M *p* < 0.001	M *p* = 0.002	M *p* = 0.045	J_0_ *p* < 0.001	J_0_ *p* < 0.001
			J_0_ *p* < 0.001	J_0_ *p* < 0.001	J_0_ *p* < 0.001	J_0_ *p* = 0.006	J_0_ *p* = 0.015	J_0_ *p* = 0.022	J_0_ *p* = 0.020	J_0_ *p* = 0.003	J_0_ *p* < 0.001		
1M	J_0_ *p* < 0.001	J_0_ *p* < 0.001	M *p* = 0.001	M *p* < 0.001	M *p* < 0.001	M *p* < 0.001	M *p* < 0.001	M *p* < 0.001	M *p* < 0.001	M *p* < 0.001	M *p* = 0.006	J_0_ *p* < 0.001	J_0_ *p* < 0.001
			J_0_ *p* < 0.001	J_0_ *p* = 0.001	J_0_ *p* < 0.001	J_0_ *p* = 0.002	J_0_ *p* = 0.004	J_0_ *p* = 0.014	J_0_ *p* = 0.007	J_0_ *p* < 0.001	J_0_ *p* < 0.001		
3M	J_0_ *p* < 0.001	J_0_ *p* < 0.001	M *p* < 0.001	M *p* < 0.001	M *p* < 0.001	M *p* < 0.001	M *p* < 0.001	M *p* < 0.001	M *p* < 0.001	M *p* < 0.001	M *p* = 0.005	J_0_ *p* < 0.001	J_0_ *p* < 0.001
			J_0_ *p* < 0.001	J_0_ *p* = 0.002	J_0_ *p* < 0.001	J_0_ *p* = 0.004	J_0_ *p* = 0.012	J_0_ *p* = 0.025	J_0_ *p* = 0.009	J_0_ *p* = 0.001	J_0_ *p* < 0.001		
6M	J_0_ *p* < 0.001	J_0_ *p* < 0.001	M *p* = 0.003	M *p* < 0.001	M *p* < 0.001	M *p* < 0.001	M *p* < 0.001	M *p* < 0.001	M *p* < 0.001	M *p* < 0.001	M *p* = 0.010	J_0_ *p* < 0.001	J_0_ *p* < 0.001
	J_45_ *p* = 0.035		J_0_ *p* < 0.001	J_0_ *p* = 0.005	J_45_ *p* = 0.003		J_45_ *p* = 0.029			J_45_ *p* = 0.015	J_0_ *p* < 0.001		
12M	J_0_ *p* < 0.001	J_0_ *p* < 0.001	J_0_ *p* < 0.001	M *p* < 0.001	M *p* < 0.001	M *p* < 0.001	M *p* < 0.001	M *p* < 0.001	M *p* < 0.001	M *p* < 0.001	M *p* = 0.021	J_0_ *p* < 0.001	J_0_ *p* < 0.001
			J_45_ *p* = 0.032	J_0_ *p* = 0.003						J_45_ *p* = 0.003	J_0_ *p* < 0.001		

Only statistically significant *p*-values are displayed in the matrix. For each visual field angle, *p*-values correspond to paired comparisons between baseline and each follow-up visit using Student’s *t*-test for related samples (*p* < 0.05). All reported *p*-values have been adjusted for multiple comparisons across eccentricities and visits using the Benjamini–Hochberg False Discovery Rate (FDR) procedure to control for Type I errors.

**Table 3:** Axial length values at different visits during orthokeratology treatment.

	PRE-OK	1M	3M	6M	12M	*p*-Value
Total group (N = 47)	24.76 ± 0.87	24.66 ± 0.90	24.72 ± 0.85	24.78 ± 0.86	24.86 ± 0.85	0.032 *
*p*-value		0.072	0.089	0.211	*p* < 0.001 *	
Group with variation in lag ^a^ N = 36	24.76 ± 0.80	24.66 ± 0.80	24.79 ± 0.79	24.82 ± 0.78	24.90 ± 0.77	0.055
*p*-value		0.216	0.025 *	0.040 *	*p* < 0.001 *	
Group without variation in lag ^b^ N = 11	24.69 ± 1.13	24.64 ± 1.13	24.50 ± 1.00	24.65 ± 1.09	24.73 ± 1.13	0.054
		0.106	0.405	0.148	0.385	

Values are expressed as Mean ± SD (standard deviation) in millimeters (mm). ^a^ Lag variation group includes patients who exhibit a decrease in lag during OK. ^b^ No lag variation group includes patients who do not exhibit a decrease in lag during OK. * *p* < 0.05: statistically significant pairwise comparison between PRE-OK and subsequent visits using paired Student’s *t*-test, after Bonferroni adjustment.

**Table 4:** Correlation analysis between axial length (AL) change (A), relative peripheral refraction (RPRE) change (B), and accommodative lag change (C) at 12 months (12M), including Pearson’s correlation coefficients (r) and corresponding *p*-values.

		A 12M	B 12M	C 12M
A 12M	r	1	0.193	−0.135
	*p*-value		0.194	0.196
B 12M	r		1	−0.066
	*p*-value			0.526
C 12M	r			1
	*p*-value			

AL change (A) was defined as the difference between AL at 12 months and baseline (PRE-OK); peripheral refraction change (B) as the difference between the RPRE at each visit and baseline; and accommodative lag change (C) as the difference between accommodative lag at 12 months and its baseline value.

**Table 5:** Correlation analysis between axial length (AL) change (A), relative peripheral refraction (RPRE) change (B), and accommodative lag change (C) POST-OK, including Pearson’s correlation coefficients (r) and corresponding *p*-values.

		A POST-OK	B POST-OK	C POST-OK
A POST-OK	r	1	0.039	−0.133
	*p*-value		0.718	0.226
B POST-OK	r		1	−0.086
	*p*-value			0.421
C POST-OK	r			1
	*p*-value			

(A) AL change was defined as the difference between AL at 1-month without wearing OK lenses (POST-OK) and baseline (PRE-OK); (B) peripheral refraction change, defined as the difference between the RPRE at each visit and baseline; and (C) accommodative lag change, defined as the difference between accommodative lag at POST-OK and baseline (PRE-OK).

## Data Availability

The data presented in this study are available on request from the corresponding author due to privacy and ethical restrictions.

## Abbreviations

The following abbreviations are used in this manuscript:

OK	Orthokeratology
AL	Axial Length
RPRE	Relative peripheral refractive error
SE	Spherical equivalent
STD	Standard
DA	Dual Axis

## Appendix A

**Table A1:** Mean values and standard deviations (SD) for the peripheral refraction component M at each retinal eccentricity and follow-up visit, according to orthokeratology lens design (CRT™ Standard (STD)).

CRT™ STD M (D)	30N			25N			20N			15N			10N			5N			0			5T			10T			15T			20T			25T			30T		
	Mean	±	SD	Mean	±	SD	Mean	±	SD	Mean	±	SD	Mean	±	SD	Mean	±	SD	Mean	±	SD	Mean	±	SD	Mean	±	SD	Mean	±	SD	Mean	±	SD	Mean	±	SD	Mean	±	SD
PRE-OK	−2.60	±	1.54	−2.79	±	1.60	−2.94	±	1.61	−2.91	±	1.56	−3.04	±	1.51	−3.03	±	1.45	−3.00	±	1.43	−3.03	±	1.47	−3.09	±	1.51	−2.97	±	1.30	−3.01	±	1.66	−2.92	±	1.63	−2.76	±	1.66
1N	−2.88	±	1.64	−2.71	±	1.62	−2.37	±	1.47	−2.43	±	1.31	−2.22	±	1.41	−2.12	±	1.22	−2.06	±	1.19	−2.27	±	1.28	−2.34	±	1.50	−2.53	±	1.33	−2.95	±	1.73	−3.02	±	1.82	−3.07	±	1.84
1W	−2.98	±	2.20	−2.48	±	2.00	−2.26	±	2.06	−1.61	±	1.19	−1.29	±	1.12	−1.31	±	1.38	−1.11	±	1.12	−1.30	±	1.08	−1.43	±	1.15	−1.84	±	1.54	−2.25	±	1.76	−2.84	±	1.88	−3.06	±	1.96
1M	−2.29	±	1.56	−2.04	±	1.36	−1.66	±	1.27	−1.31	±	1.22	−1.05	±	0.87	−0.81	±	0.81	−0.76	±	0.72	−0.82	±	0.78	−1.01	±	0.86	−1.26	±	0.83	−1.50	±	0.93	−1.89	±	1.07	−2.66	±	1.48
3M	−2.94	±	1.74	−2.48	±	1.51	−1.89	±	1.10	−1.43	±	1.11	−1.30	±	0.95	−0.97	±	0.88	−1.04	±	0.93	−1.02	±	1.14	−1.37	±	1.12	−1.56	±	1.26	−2.28	±	1.75	−2.69	±	1.84	−3.12	±	1.91
6M	−2.62	±	1.84	−2.31	±	1.62	−1.92	±	1.69	−1.37	±	1.21	−1.13	±	1.01	−0.98	±	1.00	−1.05	±	1.03	−0.98	±	1.08	−1.27	±	1.20	−1.57	±	1.19	−2.22	±	1.96	−2.37	±	1.78	−3.14	±	2.15
12M	−2.93	±	1.53	−2.83	±	1.58	−2.26	±	1.61	−2.14	±	1.87	−1.21	±	1.01	−1.07	±	0.83	−0.92	±	0.78	−1.09	±	0.83	−1.29	±	1.00	−1.66	±	1.29	−2.14	±	1.44	−2.84	±	2.00	−3.31	±	1.99
POST-OK	−3.06	±	1.85	−3.22	±	1.63	−3.33	±	1.60	−3.03	±	1.44	−3.21	±	1.46	−3.27	±	1.49	−3.33	±	1.48	−3.24	±	1.57	−3.36	±	1.65	−2.79	±	2.09	−3.15	±	1.42	−3.30	±	1.79	−3.21	±	1.86

**Table A2:** Mean values and standard deviations (SD) for the peripheral refraction component J0 at each retinal eccentricity and follow-up visit, according to orthokeratology lens design (CRT™ Standard (STD)).

CRT™ STD J0 (D)	30N			25N			20N			15N			10N			5N			0			5T			10T			15T			20T			25T			30T		
	Mean	±	SD	Mean	±	SD	Mean	±	SD	Mean	±	SD	Mean	±	SD	Mean	±	SD	Mean	±	SD	Mean	±	SD	Mean	±	SD	Mean	±	SD	Mean	±	SD	Mean	±	SD	Mean	±	SD
PRE-OK	−0.70	±	0.49	−0.51	±	0.42	−0.26	±	0.55	−0.20	±	0.40	−0.01	±	0.34	0.06	±	0.36	0.07	±	0.30	0.10	±	0.44	0.01	±	0.32	−0.20	±	0.28	−0.21	±	0.38	−0.43	±	0.40	−0.68	±	0.39
1N	−1.25	±	0.69	−0.88	±	0.64	−0.53	±	0.40	−0.33	±	0.37	−0.07	±	0.41	0.04	±	0.32	0.06	±	0.26	0.00	±	0.31	−0.05	±	0.34	−0.31	±	0.41	−0.56	±	0.53	−0.88	±	0.55	−1.12	±	0.65
1W	−1.73	±	0.92	−1.25	±	0.90	−0.94	±	0.73	−0.43	±	0.49	−0.19	±	0.36	−0.15	±	0.64	−0.03	±	0.28	−0.07	±	0.47	−0.18	±	0.44	−0.49	±	0.39	−0.77	±	0.61	−1.39	±	0.74	−1.75	±	0.95
1M	−1.70	±	0.80	−1.28	±	0.71	−1.02	±	0.48	−0.57	±	0.56	−0.20	±	0.43	−0.03	±	0.31	−0.01	±	0.30	−0.02	±	0.28	−0.10	±	0.38	−0.34	±	0.43	−0.64	±	0.61	−1.12	±	0.66	−1.72	±	0.93
3M	−1.74	±	0.79	−1.26	±	0.67	−0.76	±	0.53	−0.37	±	0.48	−0.08	±	0.53	0.05	±	0.31	0.02	±	0.40	−0.06	±	0.43	−0.18	±	0.51	−0.45	±	0.53	−0.82	±	0.82	−1.28	±	0.82	−1.72	±	0.94
6M	−1.62	±	0.94	−1.34	±	1.05	−0.87	±	1.04	−0.37	±	0.52	−0.08	±	0.45	0.01	±	0.46	0.02	±	0.38	−0.05	±	0.45	−0.14	±	0.45	−0.40	±	0.53	−0.69	±	0.92	−1.28	±	0.79	−1.78	±	1.08
12M	−1.81	±	0.86	−1.45	±	0.82	−0.95	±	0.80	−0.88	±	0.99	−0.14	±	0.43	0.03	±	0.39	0.20	±	0.30	0.09	±	0.25	0.08	±	0.31	−0.25	±	0.36	−0.63	±	0.62	−1.19	±	0.86	−1.70	±	0.95
POST-OK	−0.76	±	0.52	−0.54	±	0.41	−0.33	±	0.38	−0.13	±	0.34	0.09	±	0.37	0.19	±	0.33	0.19	±	0.26	0.21	±	0.28	0.16	±	0.27	−0.06	±	0.27	−0.20	±	0.39	−0.42	±	0.36	−0.66	±	0.39

**Table A3:** Mean values and standard deviations (SD) for the peripheral refraction component J45 at each retinal eccentricity and follow-up visit, according to orthokeratology lens design (CRT™ Standard (STD)).

CRT™ STD J45 (D)	30N			25N			20N			15N			10N			5N			0			5T			10T			15T			20T			25T			30T		
	Mean	±	SD	Mean	±	SD	Mean	±	SD	Mean	±	SD	Mean	±	SD	Mean	±	SD	Mean	±	SD	Mean	±	SD	Mean	±	SD	Mean	±	SD	Mean	±	SD	Mean	±	SD	Mean	±	SD
PRE-OK	−0.10	±	0.32	−0.10	±	0.22	−0.09	±	0.25	−0.02	±	0.26	−0.06	±	0.27	0.03	±	0.21	0.02	±	0.22	0.12	±	0.32	0.07	±	0.24	0.00	±	0.22	0.15	±	0.25	0.10	±	0.21	0.14	±	0.30
1N	−0.12	±	0.29	−0.07	±	0.33	−0.06	±	0.27	0.00	±	0.27	−0.01	±	0.28	0.05	±	0.20	0.02	±	0.24	0.05	±	0.23	0.11	±	0.26	0.09	±	0.24	0.10	±	0.34	0.13	±	0.36	0.11	±	0.35
1W	−0.05	±	0.50	−0.10	±	0.55	−0.06	±	0.51	0.01	±	0.42	−0.01	±	0.30	0.02	±	0.35	0.00	±	0.21	0.10	±	0.24	0.05	±	0.29	0.05	±	0.41	0.11	±	0.38	0.15	±	0.48	0.05	±	0.39
1M	−0.15	±	0.52	−0.05	±	0.45	−0.06	±	0.37	−0.05	±	0.41	−0.02	±	0.32	0.08	±	0.22	0.08	±	0.21	0.08	±	0.29	0.12	±	0.28	0.13	±	0.29	0.09	±	0.46	0.03	±	0.38	−0.01	±	0.41
3M	−0.08	±	0.58	−0.06	±	0.53	−0.03	±	0.46	0.03	±	0.39	0.03	±	0.33	0.05	±	0.30	0.06	±	0.24	0.10	±	0.30	0.07	±	0.34	0.03	±	0.36	0.12	±	0.50	0.00	±	0.53	0.02	±	0.65
6M	−0.35	±	0.52	−0.22	±	0.47	−0.15	±	0.52	−0.04	±	0.50	−0.09	±	0.38	0.03	±	0.36	0.05	±	0.29	0.07	±	0.30	0.15	±	0.36	0.23	±	0.45	0.30	±	0.49	0.21	±	0.50	0.33	±	0.90
12M	−0.47	±	0.83	−0.53	±	0.82	−0.35	±	0.58	−0.41	±	0.64	−0.05	±	0.39	−0.07	±	0.34	0.00	±	0.28	0.13	±	0.31	0.16	±	0.37	0.11	±	0.49	0.17	±	0.58	0.31	±	0.84	0.40	±	0.74
POST-OK	−0.18	±	0.32	−0.10	±	0.30	−0.08	±	0.31	−0.02	±	0.30	−0.01	±	0.22	0.06	±	0.21	0.05	±	0.26	0.07	±	0.21	0.04	±	0.24	0.06	±	0.20	0.14	±	0.28	0.14	±	0.25	0.14	±	0.34

**Table A4:** Mean values and standard deviations (SD) for the peripheral refraction component M at each retinal eccentricity and follow-up visit, according to orthokeratology lens design (CRT™ Dual Axis (DA)).

CRT™ DA M (D)	30N			25N			20N			15N			10N			5N			0			5T			10T			15T			20T			25T			30T		
	Mean	±	SD	Mean	±	SD	Mean	±	SD	Mean	±	SD	Mean	±	SD	Mean	±	SD	Mean	±	SD	Mean	±	SD	Mean	±	SD	Mean	±	SD	Mean	±	SD	Mean	±	SD	Mean	±	SD
PRE-OK	−2.76	±	1.71	−2.90	±	1.63	−3.02	±	1.59	−3.30	±	1.64	−3.15	±	1.58	−3.11	±	1.59	−3.17	±	1.49	−3.10	±	1.60	−3.28	±	1.66	−3.26	±	1.77	−3.02	±	1.70	−2.97	±	1.81	−2.87	±	1.81
1N	−2.88	±	1.81	−2.83	±	1.72	−2.72	±	1.53	−2.59	±	1.51	−2.38	±	1.39	−2.23	±	1.38	−2.19	±	1.29	−2.23	±	1.34	−2.24	±	1.55	−2.64	±	1.64	−2.58	±	1.63	−2.97	±	1.69	−3.09	±	2.10
1W	−3.00	±	2.00	−2.70	±	1.79	−2.16	±	1.61	−1.89	±	1.56	−1.52	±	1.27	−1.36	±	1.29	−1.35	±	1.20	−1.40	±	1.25	−1.72	±	1.22	−2.14	±	1.40	−2.33	±	1.78	−2.86	±	1.78	−3.16	±	2.02
1M	−2.70	±	1.86	−2.42	±	1.80	−1.96	±	1.56	−1.52	±	1.37	−1.17	±	1.02	−1.04	±	1.03	−0.91	±	0.97	−0.98	±	0.94	−1.26	±	1.11	−1.58	±	1.17	−2.16	±	1.33	−2.62	±	1.47	−2.96	±	1.75
3M	−2.88	±	2.00	−2.64	±	1.98	−1.91	±	1.56	−1.40	±	1.37	−1.26	±	1.18	−1.08	±	1.02	−0.87	±	0.95	−0.91	±	1.05	−1.31	±	1.38	−1.67	±	1.36	−2.11	±	1.55	−2.60	±	1.52	−3.17	±	1.66
6M	−3.04	±	1.96	−2.68	±	1.86	−2.04	±	1.74	−1.58	±	1.46	−1.27	±	1.31	−1.03	±	1.07	−1.03	±	1.07	−1.06	±	1.15	−1.29	±	1.29	−1.94	±	1.46	−2.12	±	1.76	−2.67	±	1.74	−3.20	±	1.93
12M	−3.24	±	1.88	−2.66	±	1.66	−2.38	±	1.99	−1.70	±	1.26	−1.38	±	1.43	−1.15	±	1.25	−1.11	±	1.08	−1.14	±	1.05	−1.41	±	1.61	−1.66	±	1.38	−2.25	±	1.66	−2.73	±	1.80	−3.45	±	2.02
POST-OK	−3.36	±	1.81	−3.30	±	1.61	−3.31	±	1.56	−3.27	±	1.56	−3.26	±	1.48	−3.25	±	1.36	−3.31	±	1.38	−3.25	±	1.40	−3.26	±	1.46	−3.39	±	1.70	−3.43	±	1.74	−3.40	±	1.75	−3.31	±	1.84

**Table A5:** Mean values and standard deviations (SD) for the peripheral refraction component J0 at each retinal eccentricity and follow-up visit, according to orthokeratology lens design (CRT™ Dual Axis (DA)).

CRT™ DA J0 (D)	30N			25N			20N			15N			10N			5N			0			5T			10T			15T			20T			25T			30T		
	Mean	±	SD	Mean	±	SD	Mean	±	SD	Mean	±	SD	Mean	±	SD	Mean	±	SD	Mean	±	SD	Mean	±	SD	Mean	±	SD	Mean	±	SD	Mean	±	SD	Mean	±	SD	Mean	±	SD
PRE-OK	−0.58	±	0.51	−0.34	±	0.52	−0.12	±	0.44	−0.09	±	0.37	0.16	±	0.41	0.14	±	0.30	0.19	±	0.38	0.16	±	0.31	0.07	±	0.51	−0.10	±	0.40	−0.09	±	0.50	−0.31	±	0.48	−0.55	±	0.64
1N	−0.98	±	0.75	−0.83	±	0.60	−0.46	±	0.56	−0.20	±	0.46	0.05	±	0.33	0.10	±	0.31	0.14	±	0.35	0.16	±	0.39	0.06	±	0.38	−0.23	±	0.41	−0.39	±	0.47	−0.77	±	0.56	−1.14	±	0.66
1W	−1.69	±	0.93	−1.27	±	0.80	−0.77	±	0.63	−0.46	±	0.52	−0.10	±	0.30	−0.02	±	0.27	0.03	±	0.29	0.01	±	0.35	−0.15	±	0.42	−0.40	±	0.49	−0.77	±	0.67	−1.23	±	0.78	−1.55	±	0.88
1M	−1.77	±	1.13	−1.44	±	0.92	−0.90	±	0.80	−0.51	±	0.68	−0.16	±	0.44	−0.06	±	0.34	−0.01	±	0.31	0.00	±	0.35	−0.20	±	0.46	−0.48	±	0.50	−0.88	±	0.65	−1.26	±	0.80	−1.76	±	1.02
3M	−1.86	±	0.98	−1.40	±	0.77	−0.90	±	0.66	−0.44	±	0.60	−0.23	±	0.51	−0.06	±	0.38	0.02	±	0.33	−0.04	±	0.54	−0.23	±	0.65	−0.46	±	0.57	−0.86	±	0.80	−1.32	±	0.62	−1.77	±	0.84
6M	−1.78	±	1.03	−1.39	±	1.00	−0.79	±	0.70	−0.37	±	0.50	−0.13	±	0.56	0.00	±	0.40	0.01	±	0.47	0.02	±	0.42	−0.03	±	0.49	−0.39	±	0.63	−0.77	±	0.90	−1.23	±	0.78	−1.66	±	0.84
12M	−1.74	±	0.87	−1.43	±	0.67	−0.98	±	1.21	−0.42	±	0.57	−0.05	±	0.65	0.02	±	0.59	0.07	±	0.53	0.08	±	0.48	0.10	±	0.59	−0.28	±	0.47	−0.57	±	0.64	−1.04	±	0.69	−1.62	±	0.88
POST-OK	−0.67	±	0.50	−0.37	±	0.45	−0.12	±	0.42	0.09	±	0.44	0.23	±	0.41	0.34	±	0.33	0.33	±	0.39	0.34	±	0.34	0.26	±	0.40	0.13	±	0.47	−0.04	±	0.43	−0.23	±	0.43	−0.47	±	0.47

**Table A6:** Mean values and standard deviations (SD) for the peripheral refraction component J45 at each retinal eccentricity and follow-up visit, according to orthokeratology lens design (CRT™ Dual Axis (DA)).

CRT™ DA J45 (D)	30N			25N			20N			15N			10N			5N			0			5T			10T			15T			20T			25T			30T		
	Mean	±	SD	Mean	±	SD	Mean	±	SD	Mean	±	SD	Mean	±	SD	Mean	±	SD	Mean	±	SD	Mean	±	SD	Mean	±	SD	Mean	±	SD	Mean	±	SD	Mean	±	SD	Mean	±	SD
PRE-OK	−0.17	±	0.26	−0.15	±	0.26	−0.11	±	0.27	−0.11	±	0.29	−0.08	±	0.24	−0.04	±	0.22	−0.03	±	0.21	−0.01	±	0.20	−0.01	±	0.26	−0.09	±	0.27	0.04	±	0.24	−0.02	±	0.26	0.01	±	0.27
1N	−0.12	±	0.38	−0.15	±	0.37	−0.21	±	0.46	−0.07	±	0.39	−0.11	±	0.31	−0.03	±	0.23	−0.04	±	0.30	−0.04	±	0.29	0.03	±	0.29	−0.10	±	0.34	0.01	±	0.37	−0.06	±	0.38	−0.08	±	0.43
1W	−0.21	±	0.47	−0.22	±	0.50	−0.19	±	0.39	−0.18	±	0.35	−0.11	±	0.32	−0.09	±	0.27	−0.06	±	0.25	0.01	±	0.27	0.05	±	0.34	0.03	±	0.34	0.02	±	0.47	0.00	±	0.51	−0.06	±	0.55
1M	−0.12	±	0.55	−0.16	±	0.49	−0.10	±	0.45	−0.10	±	0.38	−0.06	±	0.30	−0.02	±	0.30	−0.02	±	0.25	−0.02	±	0.27	−0.03	±	0.27	0.06	±	0.31	−0.03	±	0.47	−0.07	±	0.62	−0.08	±	0.61
3M	−0.12	±	0.69	−0.20	±	0.76	−0.18	±	0.66	−0.10	±	0.32	−0.03	±	0.42	−0.01	±	0.31	−0.01	±	0.20	0.00	±	0.31	0.01	±	0.47	−0.04	±	0.43	−0.03	±	0.51	−0.03	±	0.52	−0.14	±	0.67
6M	−0.33	±	0.48	−0.25	±	0.59	−0.13	±	0.50	−0.17	±	0.35	−0.06	±	0.35	−0.06	±	0.29	−0.01	±	0.34	0.00	±	0.39	0.03	±	0.33	−0.02	±	0.56	0.16	±	0.60	0.15	±	0.69	0.04	±	0.72
12M	−0.36	±	0.69	−0.32	±	0.59	−0.29	±	0.52	−0.28	±	0.51	−0.17	±	0.60	−0.06	±	0.35	−0.06	±	0.32	0.03	±	0.31	0.07	±	0.72	0.13	±	0.35	0.15	±	0.69	0.11	±	0.82	0.16	±	1.07
POST-OK	−0.19	±	0.30	−0.18	±	0.26	−0.13	±	0.29	−0.10	±	0.25	−0.05	±	0.22	−0.05	±	0.19	−0.03	±	0.15	0.00	±	0.19	0.00	±	0.20	0.01	±	0.26	0.10	±	0.27	0.01	±	0.29	0.07	±	0.28

**Table A7:** Mean values and standard deviations (SD) for the accommodative lag during OK.

Total group (N = 47)	0.53 ± 0.39	0.38 ± 0.31	0.35 ± 0.42	0.34 ± 0.43	0.22 ± 0.34	0.07 ± 0.42	0.15 ± 0.29	<0.001 **	0.63 ± 0.36
*p*-value		0.015 *	0.013 *	0.022 *	<0.001 *	<0.001 *	<0.001 *		
^a^ Lag variation group (N = 36)	0.67 ± 0.30	0.45 ± 0.27	0.41 ± 0.38	0.35 ± 0.42	0.21 ± 0.30	0.12 ± 0.35	0.09 ± 0.29	<0.001 **	0.62 ± 0.38
*p*-value		0.001 *	0.002 *	0.001 *	<0.001 *	<0.001 *	<0.001 *		
^b^ No lag variation group N = 11	0.05 ± 0.11	0.16 ± 0.31	0.12 ± 0.43	0.28 ± 0.45	0.24 ± 0.45	0.10 ± 0.56	0.34 ± 0.21	0.122	0.68 ± 0.30
Total group (N = 47)		0.284	0.635	0.097	0.182	0.380	0.005 *		

Total group: No significant differences were found when comparing the POST-OK value (0.63 ± 0.358 D) with the baseline PRE-OK value (0.53 ± 0.39 D) (*p* = 0.221; paired Student’s *t*-test). Note: D: diopters; SD: standard deviation. ^a^ Lag variation group includes patients who exhibit a decrease in lag during OK. ^b^ No lag variation group includes patients who do not exhibit a decrease in lag during OK. * *p* < 0.05; paired Student’s *t*-test. Comparing PRE-OK values with those from the remaining visits. ** *p* < 0.05; repeated-measures ANOVA.
